# AAV Targeting of Glial Cell Types in the Central and Peripheral Nervous System and Relevance to Human Gene Therapy

**DOI:** 10.3389/fnmol.2020.618020

**Published:** 2021-01-11

**Authors:** Simon J. O’Carroll, William H. Cook, Deborah Young

**Affiliations:** ^1^Spinal Cord Injury Research Group, Department of Anatomy and Medical Imaging, School of Medical Sciences, University of Auckland, Auckland, New Zealand; ^2^Molecular Neurotherapeutics Group, Department of Pharmacology and Clinical Pharmacology, School of Medical Sciences, University of Auckland, Auckland, New Zealand

**Keywords:** gene therapy, AAV, glia, astrocyte, oligodendrocyte, microglia, peripheral nerve, Müller glia cell

## Abstract

Different glial cell types are found throughout the central (CNS) and peripheral nervous system (PNS), where they have important functions. These cell types are also involved in nervous system pathology, playing roles in neurodegenerative disease and following trauma in the brain and spinal cord (astrocytes, microglia, oligodendrocytes), nerve degeneration and development of pain in peripheral nerves (Schwann cells, satellite cells), retinal diseases (Müller glia) and gut dysbiosis (enteric glia). These cell type have all been proposed as potential targets for treating these conditions. One approach to target these cell types is the use of gene therapy to modify gene expression. Adeno-associated virus (AAV) vectors have been shown to be safe and effective in targeting cells in the nervous system and have been used in a number of clinical trials. To date, a number of studies have tested the use of different AAV serotypes and cell-specific promoters to increase glial cell tropism and expression. However, true glial-cell specific targeting for a particular glial cell type remains elusive. This review provides an overview of research into developing glial specific gene therapy and discusses some of the issues that still need to be addressed to make glial cell gene therapy a clinical reality.

## Introduction

The term glia relates to types of non-neuronal cells in the central nervous system (CNS) and peripheral nervous system (PNS) that maintain homeostasis and are active regulators of numerous physiological functions. The glial cells of the CNS include astrocytes, which support the blood-brain barrier (BBB), provide nutrients to neurons and play a crucial role in maintaining extracellular ion balance and neurotransmitter levels in the CNS. Microglia play roles relating to both the immune response and homeostasis (Kierdorf and Prinz, 2017) and oligodendrocytes primary function is to myelinate axons and provide metabolic support (Bradl and Lassmann, 2010). The retina, which is considered part of the CNS, contains Müller glia, which like astrocytes play a role in regulating blood flow, uptake of neurotransmitters, regulation of ion levels and energy storage (Bringmann et al., 2006).

Several glial cell types play similar roles within the PNS. The gut contains enteric glia, which share many similarities with CNS glia (Grubisic and Gulbransen, 2017) and are crucial for the survival of enteric neurons. Moreover, they play a key role in homeostasis, metabolism and neurotransmission as well as gut epithelial integrity, and regulate gut motility (Ruhl et al., 2004). Schwann cells are the myelinating cells of the PNS and are involved in maintaining ionic balance and providing support to axons (Kidd et al., 2013). Satellite cells are associated with neurons in peripheral ganglia and have similar functions to astrocytes in the CNS (Hanani, 2005, 2010). As well as their role in normal physiological functions of the nervous system, glia are activated under pathological conditions and contribute significantly to disease pathology in many neurodegenerative diseases, neurotrauma, peripheral neuropathies and gut inflammation. Glial cells are therefore a potential cell target for several therapeutic approaches to treat diseases of the nervous system (Ahmed et al., 2017; Spear and Mawe, 2019; Eastlake et al., 2020).

One such approach is the use of gene therapy which employs viral vectors to deliver genetic material with therapeutic potential into a cell. Different viral vector systems have been developed to mediate gene delivery to different organ systems, including the CNS and PNS (Kantor et al., 2014). The use of viral vector gene therapy for the nervous system is appealing as many drugs cannot cross the BBB efficiently and it can overcome the need for repeated delivery of often short-acting drugs into the brain, spinal cord, retina and cochlea by allowing for a single, long-lasting intervention.

One of the most well-characterized vectors for gene therapy is derived from adeno-associated virus (AAV). These are considered the ideal for human gene therapy approaches as they are small and non-replicative, can transduce dividing and non-dividing cells, are non-pathogenic to humans and can provide long-lasting changes in gene expression (Ingusci et al., 2019). AAVs have been used to target a number of different tissue and cell types successfully within the CNS and PNS including neurons, astrocytes, oligodendrocytes, microglia, Müller glia, Schwann cells, and satellite cells (Berns and Giraud, 1996; Rabinowitz and Samulski, 1998; Xiang et al., 2018; Sargiannidou et al., 2020). A large number of clinical trials using AAV have demonstrated the relative safety of AAV gene therapy (Mastakov et al., 2002; Penaud-Budloo et al., 2018). However, to date, these trials have targeted neuronal cell types and retinal pigment epithelium in the retina. An AAV gene therapy approach has real potential for targeting of glial cells and in preclinical studies targeting of different glial cell types has been achieved (Howard et al., 2008; Hammond et al., 2017).

### AAV Vectors

Wild type AAVs are small, 4.7 kb, linear, single-stranded DNA (ssDNA) viruses in the Parvovirus family. They are composed of an icosahedral protein capsid of three types of subunit (VP1, VP2, and VP3), totaling 60 copies in a ratio of 1:1:10 (VP1:VP2:VP3). The genome consists of a *rep* gene, encoding four proteins necessary for viral replication; a *cap* gene that encodes the three capsid subunits through alternative splicing and translation from different start codons; and a third gene that encodes an assembly activating protein (AAP) which promotes virion assembly. These are flanked by inverted terminal repeats (ITRs) which are needed to direct genome replication and packaging (Samulski and Muzyczka, 2014). For therapeutic use, the *rep* and *cap* genes are removed and replaced by an expression cassette containing the therapeutic transgene under the control of a promoter and flanked by the AAV ITRs, forming a recombinant AAV (rAAV) (During et al., 2003). There are hundreds of variants of AAV, including the 11 natural serotypes; AAVs 1–11. The natural serotypes are defined by antigenically distinct viral capsids and although most were first isolated in humans, later serotypes were identified in non-human primate species, including rhesus and cynomolgus macaques (Gao et al., 2004; Mori et al., 2004).

### AAV Tropism

**FIGURE 1:**
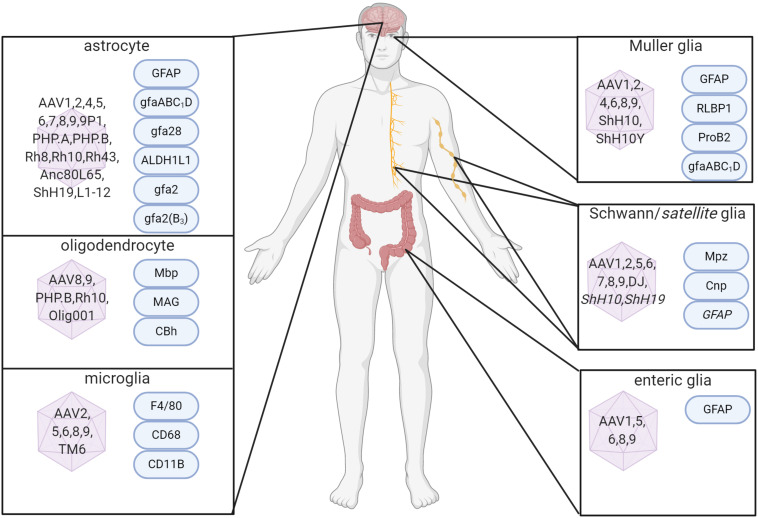
Capsid serotypes and promoters for glial targeting of AAV. Overview depicting naturally-occurring and engineered AAV viral vectors with known glial cell tropism in the CNS and PNS and relevant cell-specific promoters. References used for this figure are detailed and cited in the text. Created with BioRender.com.

In the CNS, while most AAV vectors have a preference for targeting neurons, both naturally-occurring and engineered serotypes have been shown to transduce glia (Figure 1). The tropism of an AAV for a particular cell type is dependent on the interaction of the capsid with cell surface receptors (Lisowski et al., 2015). The vector initially attaches to a cell surface glycan, which acts as a primary receptor. For efficient entry to the cell, the virus must then interact with a co-receptor. Twenty-three different glycan receptors have been identified, although the primary receptor for some serotypes has not yet been determined, whilst a number of co-receptors have also been identified (reviewed in Lisowski et al., 2015; Srivastava, 2016). AAV capsids can be modified, changing their ability to interact with specific receptors and therefore the cell types they will transduce, and this has been used successfully to change AAV tropism for a particular cell or tissue and to improve transduction efficiency.

Different strategies can be used to alter the tropism of AAV capsids (reviewed in Castle et al., 2016; Deverman et al., 2018). Chemical modification of the virus capsid can lead to improved transduction efficiency and mask native receptors allowing the vector to target alternate receptors (Bartlett et al., 1999; Ponnazhagan et al., 2002; Le et al., 2005; Carlisle et al., 2008; Horowitz et al., 2011), but these have had limited use *in vivo.* Hybrid capsids that combine the advantageous properties of specific selected AAV serotypes have been developed that lead to improved transgene expression and tropism (Koprich et al., 2010). Short peptides can also be inserted into the capsids, and their presence can allow for interaction with a specific target cell receptor (Chen et al., 2009).

Approaches can involve rational design, which is underpinned by an understanding of the function of capsid protein residues such as key residues involved in receptor binding. Mutation of these residues can lead to unique cellular tropism (Murlidharan et al., 2015), and insertion of specific peptide sequences can change cell tropism and modify the ability of the AAV vector to cross the BBB (Adachi et al., 2014; Albright et al., 2018). Another approach used to develop novel capsids is directed evolution. This involves generating highly diverse capsid libraries and using iterative rounds of selection either *in vitro* or *in vivo* to enrich for the most potent AAV variant with the desired tropism. This diversity can be created using capsid-shuffling, which involves the nuclease digestion of different AAV serotype *cap* genes that are then randomly reassembled to form chimeric genes (Koerber et al., 2009); peptide insertion, where every virus particle is engineered to display a random peptide at the capsid surface (Muller et al., 2003); or error prone PCR, which involves amplifying AAV *cap* genes in error-prone PCR reaction, with the resulting PCR products cloned to generate a diverse AAV plasmid library (Koerber et al., 2006). A more recent approach called CREATE (Cre-recombination-based AAV targeted evolution) uses Cre/lox technology to generate novel capsids and involves delivering capsid genomes containing loxP sites to animals with Cre expression in a defined cell population and then selective amplification and recovery of *Cap* sequences that transduced the target population (Deverman et al., 2016). A recent approach called BRAVE (barcoded rational AAV vector evolution), allows for large scale selection of capsids using only a single *in vivo* round of screening, unlike previous methods that require multiple rounds of enrichment (Davidsson et al., 2019). The directed evolution approach has had the greatest success in shifting AAV tropism toward certain glial cell types, and examples of this are described in the appropriate sections below.

## Astrocytes

Astrocytes play a role in several homeostatic functions within the brain and spinal cord, including controlling uptake and release of neurotransmitters, modulating synaptic activity and the supply of metabolites to neurons. Astrocytes are also components of the blood-brain barrier (BBB), where they play a crucial role in BBB integrity and function (Sweeney et al., 2019). As well as these supportive roles in normal CNS function they are well known to respond in a number of CNS disorders including Alzheimer’s disease and other aging-related dementias (Dzamba et al., 2016; Garwood et al., 2017), Parkinson’s disease (Booth et al., 2017), Huntington’s disease (Palpagama et al., 2019), Amyloid Lateral Sclerosis (ALS) (Yamanaka and Komine, 2018) and traumatic conditions such as ischemia (Rossi, 2015), spinal cord injury (Gaudet and Fonken, 2018; Okada et al., 2018), and traumatic brain injury (Burda et al., 2016).

Astrocytes respond to CNS insult by transforming their phenotype via a process called reactive gliosis. Once stimulated by injury or inflammation, several cell pathways are activated that can be either damaging or protective, many of which could be targeted as a treatment. As a result of insult or neurodegeneration, astrocytes produce molecules such as inflammatory cytokines, which activate microglia and infiltration of peripheral immune cells leading to chronic inflammation (Stephenson et al., 2018). Following traumatic injuries of the CNS, activated astrocytes migrate to the lesion where they eventually form a glial scar that produces axonal growth inhibitors, preventing axonal regeneration (Burda et al., 2016; Okada et al., 2018). Astrocyte activation leads to the loss of proper synaptic and plasticity regulation. The control of glutamatergic transmission by astrocytes is adversely affected by oxidative stress and increased production of pro-inflammatory factors (Liu et al., 2006; Santello et al., 2011). Another key role of astrocytes is in the regulation of ion flux, and disruptions to this interfere with neurotransmitter uptake by astrocytes (Djukic et al., 2007; Kucheryavykh et al., 2007). Changes in astrocytic modulation of synaptic function have been demonstrated in a model of ALS (Benkler et al., 2013). Expression of the potassium Kir4.1 channel is lost in the SOD1 mouse (Kaiser et al., 2006) and high levels of endothelin-1, which leads to activation of the AMPA receptor, is produced by activated astrocytes in this model and leads to motor neuron cell death (Ranno et al., 2014). Astrocytes produce a number of growth factors, including nerve growth factor (NGF), brain-derived growth factor (BDNF), and fibroblast growth factor (FGF) which all play an essential role in neuronal function (Miyazaki and Asanuma, 2017). Reduced growth factor levels have been associated with neurodegenerative disease. A decrease in serum BDNF levels is associated with cognitive impairment in dementias and changes in BDNF levels in the hippocampus may be linked with emotional symptoms relating to Alzheimer’s disease (Budni et al., 2015).

As well as the negative effect of reactive gliosis, astrocytes can have protective effects. For example, activating the TGF-β signaling pathway in astrocytes limits the degree of inflammation following stroke (Cekanaviciute et al., 2014). As a reaction to oxidative stress, expression of Toll-like receptor-3 is increased in astrocytes, which upregulates anti-inflammatory cytokines whilst reducing the levels of pro-inflammatory cytokines (Bsibsi et al., 2006). The interferon pathway in astrocytes is also protective. Interferon regulatory factor 3 suppresses astrocyte inflammatory cytokine gene expression following inflammatory insult (Tarassishin et al., 2011). Interferon-1 production by astrocytes is known to regulate immune responses of brain endothelial cells via anti-inflammatory effects (Rothhammer et al., 2016).

Mutations in key astrocyte genes have been associated with neurodegenerative disease. Mutations of Fyn tyrosine kinase are associated with increased inflammatory responses in Alzheimer’s disease (Lee et al., 2016, 2017). Mutations in the gene encoding TGF-β have been associated with AD risk (Caraci et al., 2012, 2018). Mutations in the astrocyte protein apolipoprotein E4 can impair amyloid-beta (Aβ) clearance (Liu et al., 2013) and may be linked to oxidative stress and inflammation (Liu et al., 2015). PARK7, a regulator of astrocyte metabolism has been found to be mutated in cases of familial Parkinson’s disease (Bandopadhyay et al., 2004), is important for astrocyte mitochondrial function and its loss leads to oxidative stress (Kumaran et al., 2007; Larsen et al., 2011). Therefore, astrocytes are a potential cell target for AAV-mediated gene therapy strategies that modulate either the inflammatory or protective effects of reactive gliosis or through potentially expressing normal copies of mutated genes expressed in astrocytes.

### AAV-Based Approaches for Targeting Transgene Expression to Astrocytes

A number of studies have looked at the tropism of AAV vectors for astrocytes. Most AAV serotypes demonstrate broad tropism without absolute specificity, but some differ in their absolute levels of transgene delivery to specific tissues. This depends on the experimental model as cell receptors for AAVs are likely expressed differently *in vitro* and *in vivo* (Royo et al., 2008). This is reflected in the variation of AAV tropism observed in each study as shown in Table 1.

**TABLE 1:** Astrocyte transduction of AAV serotypes with pan-cellular promoters.

Study	Hammond et al., 2017	Harding et al., 2006 (mouse)	Harding et al., 2006 (glioma)	Schober et al., 2016	Klein et al., 2008	Wang et al., 2003	Howard et al., 2008	Royo et al., 2008	Hutson et al., 2012	Lawlor et al., 2009	Davidson et al., 2000	Liu et al., 2005
Model	*In vivo*	*In vivo*	Xenograft	*In vivo/vitro*	*In vivo*	*In vivo*	*In vitro*	*In vitro*	*In vivo*	*In vivo*	*In vivo*	*In vivo*
Promoter	CBA	CAG	CAG	CMV	CMV	CMV	CMV	CMV	CMV	CAG	RSV	RSV
1/AAV2 ITR	++			++		++	−^	+	++			
2/2		+	++	++		+	−	++	+		+	
3/2									−			
4/2									−		−	++
5/2		++	++	++			++		++		++	
6/2		+	++	+++			−^		++			
7/2		+	++				−^	+				
8/2	++*	+	++		++		−^	++	+	+		
9/2	++*				−		−	++				
rh43/2										+++		

*, wild-type virus; ^, had transduced astrocytes 2 days after treatment. AAV, adeno-associated virus; CBA, CMV-Enhancer/Chicken β-Actin Promoter; CMV, Human Cytomegalovirus Immediate/Early Gene Promoter and Enhancer; CAG, Chicken β-Actin/Cytomegalovirus Hybrid Promoter; RSV, Rous Sarcoma Virus Long Terminal Repeat Promoter.

In primary cultures of rat CNS cells, AAV5 appears to demonstrate the strongest glial tropism under the control of a constitutively active CAG or CMV promoter (Harding et al., 2006; Howard et al., 2008). Further, AAVs 1, 2, 6, 7, 8, and especially 9 can transduce both neurons and astrocytes (Howard et al., 2008; Royo et al., 2008; Schober et al., 2016). Newer, more novel serotypes have expanded the repertoire and potential for glial transduction, but many of these have yet to be compared with the naturally-occurring serotypes (Cearley et al., 2008).

*In vivo* animal studies have demonstrated some astroglial transduction with AAVs 1, 2, 5, 6, and 8 (Davidson et al., 2000; Wang et al., 2003; Harding et al., 2006; Klein et al., 2008; Hutson et al., 2012; Schober et al., 2016; Hammond et al., 2017), but AAVrh43 has been shown to have more specific astrocyte targeting when compared to AAV8 (Lawlor et al., 2009). In a separate study, AAV4 has demonstrated strong transduction of astrocytes when injected into the brain parenchyma (Liu et al., 2005). However, this has not been replicated in side-by-side comparisons with other serotypes. AAVs rh8 and rh10, in addition to rh43 and 9 can penetrate the BBB and transduce both neurons and glial cells (Foust et al., 2009; Gray et al., 2011; Yang et al., 2014). Glial transduction was more robust in adult animals, while transduction in neonatal animals was primarily neuronal (Foust et al., 2009; Gray et al., 2011; Yang et al., 2014). This may be an example of differential receptor expression causing altered tropism, in this case between neonatal and mature adult mice. Despite the existence of certain trends, many of the studies only compare a limited number of AAV serotypes and other variables are not controlled for between studies. Even AAV purification methods have led to differences in tropism (Klein et al., 2008). This indicates that for new experiments, it is worth comparing as many serotypes as possible to ensure the best choice for a specific set of experimental conditions.

Several synthetic AAVs that have been developed to improve transduction of the CNS have demonstrated improved ability to target astrocytes. AAV9P1 is a synthetic AAV9 variant that produces selective and robust astrocyte transduction *in vitro* (Kunze et al., 2018). This vector was identified from a screen of 30 artificial AAV variants, generated by introducing specific peptides into the AAV capsid sequence of AAV1, 2, 6, 8, and 9. While this variant was shown to have relatively good astrocyte specificity *in vitro* (the transduction rate for primary human neurons was around 10%), to date no data is available on whether this specificity is still seen *in vivo*. The CREATE approach led to the discovery of a variant AAV-PHP.B, which can transduce the CNS much more efficiently than AAV9, and is able to transduce the majority of astrocytes (> 75%) in multiple CNS regions of the mouse brain (Deverman et al., 2016). However, this does not have selectivity for astrocytes as it can effectively transduce neurons and oligodendrocytes. Another variant AAV-PHP.A improved the selective targeting to more than 80% of transduced cells being ALDH1L1 + ve. However, when AAV-PHP.A and AAV-PHP.B were used to transduce human iPSC-derived cortical spheroids, only around 15% of AAV-PHP.A transduced cells were glial fibrillary acidic protein (GFAP)-positive, compared with 40% for AAV-PHP.B. There did not appear to be any difference in selectivity for astrocytes between the two variants (Deverman et al., 2016). The AAV capsid Anc80L65, developed using *in silico* reconstruction of the viral evolutionary lineage transduces astrocytes with around four times the efficacy of AAV9 (Hudry et al., 2018). A study that utilized molecular evolution to engineer novel AAV variants using directed evolution and a panel of 4 distinct AAV libraries found variants that had increased astrocyte transduction. Two AAV mutants, ShH19 and L1–12, transduced astrocytes 5.5- and 3.3-fold, respectively, compared to the parent AAV2. However, the percentage of astrocytes showing expression from these vectors was very low; 15% for ShH19 and 9% for L1–12 (Koerber et al., 2009). While these vectors described above are not astrocyte-specific, the use of these with astrocyte-specific promoters could have potential.

Most of the work to date developing astrocyte-specific promoters for gene therapy has focused on the use of the promoter for GFAP. GFAP is an intermediate filament protein that is expressed almost exclusively by astrocytes (Yang and Wang, 2015). This fact has led to its promoter being used to direct transgene activity to astrocytes, and there is a large amount of literature that shows this can be achieved. Many studies have tested the incorporation of GFAP promoters to drive astrocytic-specific expression using viral vectors. Transgene expression under the transcriptional control of the 2.2 kb human *GFAP* promoter, gfa2, has been shown to be expressed in astrocytes throughout the brain (Lee et al., 2008). However, as with many cell-type-specific promoters, the large size of this promoter has severe limitations when used with AAV vectors, due to it occupying a considerable amount of the vector genome. Different strategies have been employed to shorten the *GFAP* promoter to make it more suitable for use in AAV vectors. An AAV vector containing a truncated 448 base-pair gfa28 promoter (Lee et al., 2006, 2008), was able to drive gene expression much more strongly than the full-length promoter when tested *in vitro*. However, when this promoter was used *in vivo* in mice, the level of transgene expression driven by this promoter was comparable to the full-length gfa2 promoter, expression was restricted to certain CNS regions, and neuronal expression was observed as well as in astrocytes. Based on this finding Lee et al. (2008) created transgenic mice with promoters containing different enhancer fragments to determine which were required to silence neuronal signaling, and to restrict expression to specific brain regions. This work led to the discovery of a 681 bp *GFAP* promoter, gfaABC_1_D, which exhibited mostly the same expression pattern in the brain as the full-length 2,210 bp gfa2 promoter but had a twofold greater expression that was largely restricted to astrocytes (Lee et al., 2008). Similarly a 681 bp gfaABC_1_(mC_1__.__1_)D variant had expression limited to astrocytes in the dorsal and caudal cortex, hippocampus and caudal vermis of the cerebellum. This study demonstrates that it may be possible to further limit gene expression to specific glial populations by modifying cell-specific promoters (Lee et al., 2008). de Leeuw et al. inserted additional copies of the *GFAP* enhancer regions to determine if these would increase its transcriptional activity. Injection of an adenoviral construct containing the gfa2 promoter engineered to contain three copies of the B enhancer region [gfa2(B_3__)_] resulted in greater gene expression in astrocyte cell cultures and expression that was limited to GFAP-positive cells when injected into the basal ganglia of mice (de Leeuw et al., 2006). However, again due to its size, there are issues in using this in the context of AAV vectors.

A number of studies have used AAV vectors containing the 681 bp gfaABC_1_D with the goal of obtaining astrocyte specificity (Xie et al., 2010; Theofilas et al., 2011; Dirren et al., 2014; Dvorzhak et al., 2016; Vagner et al., 2016; Taschenberger et al., 2017; Griffin et al., 2019, 2020; Testen et al., 2020). While several studies show good evidence of astrocyte specificity (Xie et al., 2010; Theofilas et al., 2011), other studies report transduction of other cell types (Taschenberger et al., 2017; Griffin et al., 2019). For instance, we observed transgene expression in lower motor neurons but not in neurons of the dorsal horn following vector infusion in the adult rat spinal cord (Griffin et al., 2019). Very high levels of transgene expression in lower motor neurons was also reported with the full-length GFAP promoter (Peel and Klein, 2000). One approach that has been used to overcome the issue of lack of astrocyte specificity is the incorporation of cell-specific microRNAs (miRNAs) to suppress off-target transgene expression in particular cell types (Brown et al., 2006). Endogenous expression of miR124, which is specific to neurons is able to repress gene expression in neuronal cells (Colin et al., 2009) and addition of miRNA recognition sequences to viral constructs can suppress leaky gene expression from AAV vectors (Shimizu et al., 2014). When target sequences for miR124 were included in the 3′ UTR of an AAV expression plasmid containing a transgene under the control of the gfaABC_1_D promoter, neuronal transgene expression in the rat striatum was completely absent compared to around 10% neuronal expression with the gfaABC_1_D promoter alone (Taschenberger et al., 2017). However, the presence of the miR sequence strongly reduces the number of astrocytes expressing the transgene to around 10% of that seen with the gfaABC1D promoter alone, calling into question the usefulness of this approach for improving astrocyte specificity.

One issue with the use of a GFAP promoter is that the levels of GFAP expression can be variable in different parts of the CNS and relatively low in some brain regions (Hajos and Kalman, 1989; Kalman and Hajos, 1989). Therefore using this promoter may not always be appropriate. Aldehyde dehydrogenase family 1, member L1 (ALDH1L1) has been characterized as a pan-astrocytic marker that is found more homogeneously throughout the brain than GFAP (Cahoy et al., 2008). Mudannayake et al. (2016) tested several different AAV serotypes under the control of a putative rat *Aldh1l1* promoter for astrocyte selectivity in the rat substantia nigra pars compacta (SNpc) brain region and found transgene expression was exclusively expressed in neurons and independent of AAV serotype used. Neuronal-specific transgene expression was also found following intrahippocampal vector infusion, but expression was found in both neurons and astrocytes in the striatum following intrastriatal vector infusion. In a later study by Koh et al. (2017) using a human *ALDH1L1* promoter, an AAV-hALDH1L1-Cre vector was injected into several brain regions of the Ail4 (RCL-tdTomato) mouse, found tdTomato gene expression was also seen to be predominantly neuronal in most brain regions analyzed. Interestingly, in the thalamus, this expression pattern was reversed, with the majority of tdTomato expression found in astrocytes (92%) with minimal neuronal expression (2%). Therefore, the use of the ALDH1L1 promoter may have the potential for targeting astrocyte expression in the thalamus, especially as GFAP expression in this region appears to be very low (Kalman and Hajos, 1989).

Other potential astrocyte-specific gene promoters have also been suggested (Kery et al., 2020) including *Slc1a3*, which codes for the glutamate transporter SLC1A3 (also known as GLAST or EAAT1) (Sery et al., 2015). A 636 bp region 5′ upstream of the gene can drive strong gene expression, and so this relatively small promoter might have potential use in AAV vectors (Hagiwara et al., 1996). Another potential promoter is *Gjb6*, which codes for the gap junction protein Connexin30. Connexin30 is only expressed in gray matter astrocytes and so this promoter could be used to specifically target these populations (Nagy et al., 1999; Sohl et al., 2004).

## Oligodendrocytes

Oligodendrocytes are the myelin-producing cells of the CNS. This myelin forms an insulating membrane that wraps tightly around axons that allows for rapid signal conduction and is crucial for normal CNS function (Kuhn et al., 2019). Oligodendrocytes and the myelin sheath also provide trophic support for axons, such as the production of neurotrophic factors (Bradl and Lassmann, 2010) and lactate that is passed to axons to partake in the metabolic pathways involved in producing ATP (Bercury and Macklin, 2015). Oligodendrocytes are particularly sensitive to excitotoxic and cytotoxic factors and damage of the CNS. The high metabolic rate required for myelination and the presence of high levels of iron, which is required as a co-factor for this process, can lead to high levels of reactive oxygen species, free radical formation and lipid peroxidation. This combined with the presence of low levels of the antioxidant enzyme glutathione in oligodendrocytes makes this cell type particularly sensitive (Bradl and Lassmann, 2010). Oligodendrocyte pathology is, therefore, present in a range of CNS disorders (Fern et al., 2014). This includes leukodystrophies, which are a group of inherited disorders that lead to white matter degeneration (Vanderver et al., 2015), multiple sclerosis (Procaccini et al., 2015), Alzheimer’s disease (Nasrabady et al., 2018), Parkinson’s disease (Bohnen and Albin, 2011), Fragile X syndrome (Filley, 2016), ischemic stroke (Wang et al., 2016), spinal cord, and traumatic brain injury (Fern et al., 2014; Hassannejad et al., 2019; Pukos et al., 2019), as well as in conditions such as schizophrenia and depression (Wang et al., 2014; Najjar and Pearlman, 2015). Gene therapy approaches have the potential to protect against toxicity or to promote remyelination.

### AAV-Based Approaches for Targeting Transgene Expression in Oligodendrocytes

No natural AAV capsid that exhibits primary oligodendrocyte tropism has been described. While a small number of serotypes can transduce this cell type when combined with pan-cellular markers, the overall transduction efficiency is low (Lawlor et al., 2009). These include AAV8 and 9 when paired with a cytomegalovirus (*CMV*) promoter (Hutson et al., 2012; Bucher et al., 2014) and AAV8 with a chicken β-actin (*CBA*) promoter (Gray et al., 2010). In a preclinical study of metachromatic leukodystrophy, AAVrh10 has been found to transduce oligodendrocytes when driven by a cytomegalovirus/β-actin hybrid (*CAG/cu*) promoter (Piguet et al., 2012). AAV-PHP.B’s widespread transduction of the mouse CNS includes oligodendrocytes when using a *CAG* promoter (Deverman et al., 2016). DNA shuffling and directed evolution approaches have also produced a chimeric capsid that transduced both neurons and oligodendrocytes (Gray et al., 2010), with a novel AAV capsid shown to have excellent oligodendrocyte preference (Powell et al., 2016). The Olig001 vector, which contains a chimeric mixture of AAVs 1, 2, 6, 8, and 9 had a > 95% specificity for oligodendrocytes (as assessed by GFP expression) following striatal vector infusion into rats even though transgene expression was under the control of the CBA promoter. The other 5% of cells transduced were neurons, and no astrocyte or microglial expression was seen.

In order to specifically target transgene expression to oligodendrocytes, a number of cell-specific promoters have been trialed. Initial studies used the promoter from the gene for myelin basic protein (MBP), which is a major constituent of the myelin sheath of both oligodendrocytes and Schwann cells. A 1.9 kb *Mbp* promoter was able to drive GFP expression in the MOCH-1 transformed oligodendrocyte cell line and primary rat oligodendrocyte cultures (Chen et al., 1998). In primary cultures, GFP expression was almost exclusively in oligodendrocytes, although some expression was observed in astrocyte-like cells. When AAV vector was injected into the cerebral hemisphere of mice, GFP expression appeared to be only in oligodendrocytes and was not seen in astrocytes, microglia or neuronal filaments. Building on this work, the authors then investigated cell and tissue specificity and the duration of transgene expression following injection of the vector into different regions of the mouse brain (Chen et al., 1999). High-levels of GFP expression were almost exclusively seen in white matter areas of the brain with very limited expression in areas of gray matter. When cell-specificity was determined based on morphology, anatomic location, and cell-type specific immunohistochemistry, GFP expression was found to be almost exclusively in oligodendrocytes with no expression seen in neurons, astrocytes or microglia.

While this data suggests that cell-specific promoters such as those for MBP can be used to target oligodendrocytes specifically, it is also important to determine how the stage of development would impact this. This is essential to understand when using somatic gene transfer approaches for glia in the developing brain to treat genetic conditions such as leukodystrophies. von Jonquieres et al. (2013) tested this by injecting a chimeric AAV 1/2 vector expressing GFP under the control of the *Mbp* promoter into the striatum of mice at postnatal day 0 (P0) (neonates), P10 and P90 (adults). While in the P10 and P90 animals, the majority of GFP staining was localized to oligodendrocytes, for the P0 animals only around 25% of GFP + ve cells were oligodendrocytes, with the majority (56%) being astrocytes and the number of oligodendrocytes transduced was very low at only 3%. While for the P10 and P90 animals the majority of transduced cells were oligodendrocytes, there was a different pattern in the degree of transduction of other cell types. In the P90 animals, around 20% of transduced cells were neurons, and no GFP was detected in astrocytes. However, in the P10 animals, no GFP was detected in neurons, but it was seen in astrocytes, although in small numbers (3.6% of GFP + ve cells). Perhaps most interestingly at P10, transduction was almost exclusively in oligodendrocytes (96%), compared with around 75% in the adult mice. The authors suggested that this vector could allow for treatment of developmental gliopathies as brain development in the P10 mouse corresponds to that seen at the beginning of the last trimester in human pregnancy (Clancy et al., 2001). When the vector was injected into the brains of P10 homozygous ASPA^*lacZ/lacZ*^ mice, which are a model of the early onset leukodystrophy Canavan disease, a similar pattern of oligodendrocyte specificity was seen as in WT mice (von Jonquieres et al., 2013). While this is promising for the potential to treat developmental gliopathies such as leukodystrophies, due to the marked difference in cell expression patterns seen between P0 and P10 animals, much more work would be required to understand changes in expression during human development before it could be used for such a purpose. In a subsequent paper these authors looked at transduction of non-chimeric AAV variants rh20, rh39, and cy5 and their ability to drive expression under the control of the *Mbp* promoter, in the striatum of adult mice. They showed that oligodendrocyte specificity was greater for rh39 (91%) and cy5 (87%) when compared to AAV 1/2 (78%) (von Jonquieres et al., 2016).

While the *Mbp* promoter seems to have potential for oligodendrocyte targeting, its relatively large size and the poor oligodendrocyte specificity following early neonatal vector delivery (von Jonquieres et al., 2013) would limit its usefulness. However, MBP-driven transgene expression from an AAV vector has been shown to be effective in a model of oligodendrocyte disease. The Cx32/Cx47 double-knockout mouse is a well-characterized model of hypomyelinating leukodystrophy-2. An AAV vector containing the *Gjc2/Cx47* gene under the *Mbp* promoter was delivered to the internal capsule of P10 animals. This resulted in greater survival and significant motor improvement, improved myelination and reduced oligodendrocyte apoptosis, inflammation and astrogliosis (Georgiou et al., 2017).

The myelin-associated glycoprotein (MAG) has been tested for its ability to drive oligodendrocyte-specific expression using AAV (von Jonquieres et al., 2016). MAG is a protein responsible for recognition of axons and maintenance of myelin. Based on an *in silico* analysis of the MAG promoter, the authors generated AAV plasmids expressing GFP under the control of either a 2.2 kb *MAG* promoter or truncated 1.5 and 0.3 kb fragments. All three AAV constructs were packaged into cy5 vectors and injected into the striatum of adult mice. All three constructs showed very good oligodendrocyte specificity, with GFP expression almost exclusively confined to oligodendrocytes, with between 98.4% for the 2.2 kb to 90.7% for the 0.3 kb promoter. The percentage of oligodendrocytes transduced was 65, 82, and 57% for the 2.2, 1.5, and 0.3 kb promoters, respectively. When the vector containing the 2.2 kb promoter was injected into the brains of P0 pups, the specificity for oligodendrocytes was seen to be around 80% in comparison to the *Mbp* promoter where only around 25% specificity for oligodendrocytes was seen, suggesting use of the *MAG* promoter may be better suited to treatment of developmental gliopathies. However, the authors did not test the smaller promoters. The specificity and percentage of oligodendrocytes transduced appeared to be less for the smaller promoters. It would have been interesting to look at their profile in P0 pups, especially as the 0.3 kb promoter is likely to be the most useful for use with AAV vectors due to its small size.

A recent report by Powell et al. (2020) demonstrated that modification of constitutive promoters can shift gene expression from neurons to oligodendrocytes. Infusion of an AAV9 vector with a full-length *CBA* promoter into the rat striatum led to predominantly neuronal (88.4%) transgene expression. However, the use of a truncated CBA promoter (*CBh*) showed only 46% of neurons but 38% of oligodendrocytes were labeled. When an AAV2 vector was used, expression was predominantly neuronal for both promoters. This suggests that certain AAV capsids can influence promoter activity between different cell types. When six glutamate residues were inserted into the VP2 region of the AAV9 capsid, this shifted the transgene expression profile from the full-length promoter from neurons to oligodendrocytes (80%). However, when these amino acids were changed in AAV2, no change in expression was seen. This ability for capsid sequence to influence gene expression could be used to target particular cell types.

## Microglia

Studies have tested various AAV serotypes for their ability to transduce microglia, and the level of transduction is generally low (Maes et al., 2019). In early studies, Bartlett et al. (1998) found that while AAV2 was able to transduce microglia, as determined using Cy3-labeled virions, no transgene expression was observed. Similarly, no transgene expression was observed in primary microglia cultures following application of AAV1–9 or rh10 vectors (Rosario et al., 2016) although others showed 80% of the cells expressed GFP when AAV2-CMV-eGFP was applied to primary neonatal and adult microglia (Su et al., 2016). When different serotypes (AAV2, AAV5, AAV6, AAV8, and AAV9) were applied to cultured neonatal microglia, AAV6-CMV-eGFP produced an 80-fold increase in transgene expression compared to AAV2-CMV-eGFP while AAV8 resulted in a 25-fold increase in transgene expression. However, when the expression of microglial M1 and M2 activation markers was assessed to determine the effect of viral vector-mediated transduction and/or transgene expression, AAV6 and AAV9 elevated expression of the scavenger receptor MARCO, while the M2 phenotype marker YM1 was down-regulated in cells treated with AAV5 and AAV8. AAV2 did not produce any significant change in any activation markers assessed or influence the phagocytic activity of the microglia (Su et al., 2016). Therefore, development of an AAV to target microglia *in vivo* will require an understanding of the effect a serotype will have on the activation state of microglia, to prevent any unwanted, potentially detrimental inflammatory side-effects of the treatment.

Several macrophage-specific promoter sequences, human *CD11B* and *CD68*, and murine *F4/80*, have been assessed for their ability to provide specific microglial targeting both *in vitro* and *in vivo*. When AAV2 and AAV5 vectors expressing transgenes under control of these promoters were applied to rat primary microglia cultures, transduction efficiencies of around 25% for *F4/80*, 10% for *CD68*, and only one cell per field-of-view for *CD11B* were reported for both vectors. While these transduction efficiencies are low, transgene expression did appear to be microglia-specific as no expression was detected following transduction of rat primary neuronal cultures (Cucchiarini et al., 2003). When AAV5 vector containing the *F4/80* promoter was injected in to brains of adult rats, transgene expression appeared largely localized to F4/80-labeled cells.

Modified AAV capsids have also shown some success in improving transduction of microglia. Rosario et al. modified the AAV6 capsid (AAV6) through site-directed mutagenesis of two tyrosine residues to phenylalanine and a threonine to valine (Y731F/Y705F/T492V), which has been shown to increase transduction efficiency in monocyte-derived dendritic cells (Pandya et al., 2014). The combination of this modified AAV capsid and either the *F4/80* or *CD68* promoter resulted in > 95% transduction of microglia and no neuronal or astrocytic expression in mixed glial or primary microglial cultures. When tested *in vivo*, microglial specificity was shown to be around 75% for F4/80 and 20% for CD68 following injection into P0 rat pups and the adult rat hippocampus, although the total numbers of microglia showing expression appeared to be low (Rosario et al., 2016).

An important consideration is the impact that the activation state of microglia will have on expression from specific vectors used. For example, levels of CD68 are greater in pro-inflammatory microglia, which could explain the low level of expression seen in a healthy brain. This expression pattern could potentially be of use when targeting activated microglia, where it is likely that gene expression would be highest in this population of cells.

## Müller Cells

Müller glial cells are a major cellular component of the retina and engage in numerous roles vital to retinal function, such as structural, nutritional, homeostatic, osmotic, metabolic, and growth factor support to retinal neurons (Bringmann et al., 2006; Reichenbach and Bringmann, 2013). These glial cells also interact with blood vessels and are involved in the function of the blood–retina barrier and regulating retinal blood flow (Newman, 2015). In response to retinal injury, stress, or degeneration, Müller glial cells undergo active gliosis (Graca et al., 2018). A number of diseases including diabetes, macular edema, and ischemia lead to hypertrophy and hyperplasia of the Müller glia that contributes to a chronic inflammatory retinal environment and ultimately cell death (Coughlin et al., 2017). It has been demonstrated that Müller cells have a neuroprotective phenotype. For example, they respond to disease or injury-mediated photoreceptor stress by upregulating secretion of neurotrophic factors such as basic fibroblast growth factor (bFGF) and ciliary neurotrophic factor (CNTF) (Greenberg et al., 2007). Mutations in several genes expressed in Müller glial cells have been linked to retinal dystrophies (Maw et al., 1997; Zhao et al., 2015). Therefore Müller glial cells have been proposed as targets for therapeutic approaches to retinal diseases such as gene replacement therapy or boosting neurotrophin secretion to enhance their neuroprotective properties (Dorrell et al., 2009; Pellissier et al., 2015). As these cells transverse the entire thickness of the retina, they would have the ability to facilitate expression of factors throughout all layers of the retina (Reichenbach and Bringmann, 2013).

A number of different AAVs have been shown to have tropism for Müller cells. When AAV 1, 2, and 5 were delivered to the retinas of E13 mice via subretinal injection, only AAV1 and 2 transduced Müller cells (Surace et al., 2003). Intravitreal injection of AAV 1, 5, 8, and 9 showed occasional Müller cell transduction in 2 month-old C57BL/6 mice (Lebherz et al., 2008). Subretinal injection of AAV8 and 9 also shows Müller cell transduction in 4 week-old mice, but transduction was not seen with AAV5 (Allocca et al., 2007). When retinal transduction by AAV2 was compared following intravitreal injection into P0, P14 and adult mice, transduction was seen in Müller cells at all ages with highest levels of transduction observed in adults, where around 10% of AAV transgene expression was observed (Harvey et al., 2002). AAV4 and 6 also have tropism for Müller cells (Hellstrom et al., 2009). In all these cases the number of Müller cells transduced was low, compared with other retinal cell populations. However, Pellissier et al. (2014) reported that use of AAV9 vectors led to transduction of around 30% of Müller cells along with photoreceptor cells and retinal pigment epithelia. These authors used AAV9 under the control of a CMV promoter to deliver the Crumbs-homolog-2 (CRB2) protein, which is expressed in Müller cells and photoreceptors, to the retinas of *Crb2* and *Crb1Crb2*^*F*/+^conditional knock-out mice, which are both models of severe, progressive retinal degeneration (Pellissier et al., 2015). This led to improved retinal function and morphology compared to untreated animals, demonstrating the potential for AAV to target and modify Müller cell-related gene mutations.

As Müller cells express GFAP, this has been used to target transgene expression to these cells. AAV-mediated delivery of neurotrophic factors, under control of the *GFAP* promoter has been shown to be protective in animal models of retinal disease. A mouse model that has a defective gene for the VLDL receptor (VLDLR) shows excessive retinal neo-vascularization (NV), which is associated with common causes of vision loss (Klein et al., 2004). The retinal phenotype of these animals is similar to that seen in human patients with certain retinal NV diseases, including glial abnormalities as evidenced by increased Müller cell activation associated with the area of NV. In order to target activated Müller cells as a potential therapy, Dorrell et al. (2009) tested an AAV2 vector containing GFP under the control of a minimal 0.35 kb human *GFAP* promoter consisting of the A/B and D sequences (Besnard et al., 1991). When the vector was delivered by intravitreal injection to wild-type mice, limited GFP expression was noted in Müller cells while in *Vldlr^–/–^* mice, a greater number of Müller cells expressed GFP, and this was also associated with transgene expression in processes adjacent to areas of NV. GFP expression appeared to be Müller cell-specific, unlike non-specific expression mainly localized to ganglion cells that was found using a non-selective *CAG* promoter. The authors then used this system to deliver neurotrophin-4 (NT-4), which is known to protect neurons in models of retinal degeneration. Using an AAV-GFAP-NT4 vector, a similar expression pattern of NT-4 as found for GFP was observed, and this was associated with protection of the retina from neuronal degeneration.

A number of AAV variants have shown enhanced targeting for Müller cells. Based on work which determined novel AAV capsids that more efficiently transduced both primary human astrocytes *in vitro* and rat astrocytes *in vivo* (Koerber et al., 2009) and the shared properties between astrocytes and Müller cells, Klimczak et al. (2009) explored the effectiveness of a number of AAV variants for their potential for intravitreal transduction of Müller cells. Compared to very low transduction from the parent AAV6, one novel variant named ShH10 demonstrated a striking increase in both transduction efficiency and specificity for Müller cells. ShH10 was able to produce diffuse expression throughout the retina with approximately 94% of transduced cells being Müller cells, with limited transduction in interneurons (2%), and retinal ganglion cells (4%) compared with approximately 76% of transduced cells being Müller cells, 3% interneurons, and 21% retinal ganglion cells for the related AAV2 virus. ShH10 was also more efficient at transducing Müller cells, transducing 22 vs. 14% of total Müller cells compared with AAV2. These authors then extended to testing the ability of the ShH10 vector to deliver GDNF to the retina via Müller cells, and its ability to modulate retinal degeneration in the S334-4ter rat model of retinitis pigmentosa (Dalkara et al., 2011). To optimize GDNF production by improving the transduction capability of the vector, they modified ShH10 via a tyrosine to phenylalanine amino acid change to create ShH10.Y445F. Mutations in conserved tyrosine residues in AAV capsids have been shown to enhance vector transduction including in the retina (Zhong et al., 2008; Petrs-Silva et al., 2009). This vector led to transduction of approximately 50% of Müller cells in the retina of TgS334 rats. This was over 100% greater transduction than that seen in WT rats, demonstrating that this mutation allows for a greater transduction efficiency, although the authors also acknowledge that the degenerating retina may be a more permissive environment for AAV transduction. When this vector was used to deliver a GDNF transgene in the diseased retina, they were able to demonstrate long-term expression of therapeutic and safe levels of GDNF for up to 5 months, and this was accompanied by a slowdown in retinal degeneration, as assessed by electroretinogram and histology. Interestingly, when the ShH10Y vector was used to specifically target Müller cells in the *Crb2* and *Crb1Crb2*^*F*/+^ models described above, no improvement in retinal function was seen (Pellissier et al., 2015), as opposed to AAV9, which targeted Müller cells and photoreceptor cells and led to functional improvement. This demonstrates that depending in the disease, specific targeting of just one cell type with an AAV may not be appropriate. These vectors combined with the use of cell-specific promoters have been used in an attempt to further improve Müller cell specificity (Pellissier et al., 2014). Pellissier et al. (2014) looked at the transduction profile of AAV6 and the two AAV6-derived variants ShH10 and ShH10Y via injection into the vitreous of *Crb1^–/–^* mice, a model of retinal dystrophy. The ShH10Y variant had an enhanced ability to transduce Müller cells, with 2–3 times the transduction efficiency of the other vectors. This supports the idea that the increase in transduction seen by Dalkara et al. (2011) is due to the vector modification and not the diseased environment. The authors tested ShH10Y vectors with GFP expression under the control of a full-length (1.8 kb) or minimal (0.4 kb) *Cd44* promoter or the 2.8 kb *RLBP1* promoter (Pellissier et al., 2014). However, the *Cd44* promoters showed only low levels of expression, that was not restricted to Müller cells. Conversely, the *RLBP1* promoter caused high levels of expression that was restricted to Müller cells. However, due to its size, a shortened promoter would likely be required for it to be of practical use for expression of most genes. More recently Cao et al. (2020) found using an AAV2/6 mutant rAAV2/6-S663L led to increased tropism for Müller cells when injected intravitreally into 5 week-old mice and when gene expression as driven by the *GFAP* promoter that gene expression was specifically seen in Müller cells.

Due to the fact that natural “cell-specific” promoters often target expression to more than one cell type, attempts have been made to screen a library of promoters driving transgene expression in regions of interest. Jüttner et al. developed a library of 230 AAVs, each with a different synthetic promoter and tested their transduction in the retina of mice, non-human primates and humans (Juttner et al., 2019). This screen found a number of synthetic promoters that targeted Müller cells with 100% specificity and one in particular (labeled ProB2) transduced 45% of Müller cells.

Attempts have been made to create vectors that will restrict gene expression to an injured retina. Hypoxia in the eye is known to be a causative factor in a number of retinal diseases, such as diabetic retinopathy and age-related macular degeneration (Campochiaro, 2015). When an AAV2 vector containing a hybrid promoter consisting of the GfaABC_1_D promoter and hypoxia-responsive and aerobically silenced elements (HRSE) (Wenger, 2002) was injected into a mouse model of oxygen-induced retinopathy, high levels of gene expression was seen in Müller cells of damaged eyes but was completely absent in mice exposed to normoxia (Prentice et al., 2011). This approach would allow for gene expression only in regions of hypoxia, which could be beneficial in reducing off-target effects of gene expression in healthy tissue.

## Peripheral Nerve

### Schwann Cells

Schwann cells are the major glia of the PNS. Two types of Schwann cells are found; myelinating, which form a myelin sheath around peripheral axons and non-myelinating, which are involved in maintaining ionic balance and providing support to axons (Kidd et al., 2013). Many neuropathies are related to mutations or inflammation of immune cells (Martini and Willison, 2016). Charcot-Marie-Tooth (CMT) disease, or non-syndromic inherited peripheral neuropathy, is one of the most common neurogenic disorders. Clinical characteristics include distal weakness, sensory loss and deformities of the feet. It is a genetically heterogeneous set of disorders with over 100 different genes implicated in disease causation. Mutations can occur in myelinating Schwann cells, and these cause demyelinating forms of neuropathy (Laura et al., 2019; Sargiannidou et al., 2020). A number of gene therapy approaches using lentiviral vectors for gene replacement strategies in models of CMT have been trialed and demonstrated promise (Eggers et al., 2013; Allodi et al., 2014; Sargiannidou et al., 2015; Kagiava et al., 2016). To date, only a few studies have looked at the feasibility of using AAV vectors to target Schwann cells in CMT.

Several AAV vectors demonstrate tropism for Schwann cells. Hoyng et al. (2015) tested AAV 1–9 for their ability to transduce cultured primary rat and human Schwann cells and rat and human nerve segments. This study showed a few differences in tropism between the species and between cells and nerve segments. AAV1 was the most efficient at transducing rat Schwann cells, with twice the number of transgene-expressing cells compared to any other AAV tested. In human Schwann cells, AAV2 and AAV6 were seen to perform equally well. However, a different transduction pattern was seen in nerve segments. AAV 1, 5, 7, and 9 were all equally successful in transducing rat nerve segments, whereas AAV2 was superior in human nerve segments. More recently, AAV1, AAV2, AAV6, and AAV-DJ were found to efficiently transduce primary human Schwann cells, with levels of transgene expression for AAV6 and AAV-DJ being 2–3 times that seen in AAV1 or 2 (Bai et al., 2019). A study by Homs et al. (2011) to determine whether AAV vectors could specifically target Schwann cells found that sciatic nerve injection of AAV8 led to specific Schwann cell expression with limited (< 1%) neuronal gene expression, unlike the CNS where significant neuronal tropism is seen. This could be explained by differences in the expression of receptors between the CNS and PNS (Homs et al., 2011).

Due to the presence of common transcription binding elements, a number of oligodendrocyte promoters are also able to drive gene expression in Schwann cells. However, the size of these promoters precludes their use in AAV vectors. A full-length *Mbp* promoter is capable of driving expression in Schwann cells but the shorter 1.3 or 1.9 kb fragments shown to drive expression in oligodendrocytes do not contain the enhancer elements required for Schwann cell expression (Mathis et al., 2000; Forghani et al., 2001). The 2,3-cyclic nucleotide (*Cnp*) and proteolipid protein (*Plp*) promoters have been shown to be expressed in Schwann cells, and a *Cnp* promoter has been used to drive expression in oligodendrocytes using lentivirus, but are again too big to use with AAV (Kagiava et al., 2014; Schiza et al., 2015). The 2.2, 1.5, and 0.3 kb fragments of the *MAG* promoter should drive expression in Schwann cells, but this has not been tested. These promoter fragments contain an RNF10 site which has been suggested would allow transgene expression in Schwann cells (von Jonquieres et al., 2016). The myelin-specific myelin protein zero (*Mpz*) promoter has been shown to have high selectivity for Schwann cells. This along with its relatively short length (1.1 kb) (Messing et al., 1992) and its successful use in targeting Schwann cells using lentivirus (Sargiannidou et al., 2020), suggests it is likely to be a good candidate for use with AAV vectors.

### Satellite Cells

Another glial cell type found in peripheral nerves are satellite cells. These are associated with neurons in the sensory, sympathetic and parasympathetic ganglia and are thought to play similar functions to astrocytes in the CNS (Hanani, 2005, 2010). Following nerve injury, satellite cells become activated, leading to chemokine/cytokine release (Ohara et al., 2009; Souza et al., 2013). This activation is an important component of pain signaling, and dysregulation can lead to chronic pain (Gosselin et al., 2010; Ji et al., 2013). Modulation of satellite cells can alter the pain responses after nerve injury, and genetic manipulation of satellite cells has been proposed as a potential treatment for pain control (Jasmin et al., 2010). A recent study tested different AAV vectors for their ability to transduce satellite cells. AAV6 as well as AAV shH10, and AAV shH19, which have been shown to have a preference for transduction of Müller glia in the retina were used and injected into the dorsal root ganglion (DRG) of adult rats. Strong expression that was restricted to neurons was observed when transgene expression was under the control of a *CMV* promoter. Conversely, when expression was driven by a *GFAP* promoter, expression was almost exclusively in satellite cells (Xiang et al., 2018). Interestingly the AAV shH10 vector, the novel capsid variant of AAV6 that demonstrates almost exclusive glial tropism in the retina, was almost exclusively neuronal on the DRG. This suggests that cellular receptors for different AAVs can vary between different glial populations in the CNS and PNS.

## Enteric Glia

The gut contains its own nervous system, termed the enteric nervous system (ENS) which regulates gastrointestinal functions such as motility, local blood flow, transport of molecules across the mucosa and modulates endocrine and immune functions (Costa et al., 2000). It consists of two interconnected ganglionated plexuses that surround the digestive tract. As well as enteric neurons, the ENS contains enteric glia, which are present in numbers up to 6 times higher than the number of neurons. These glia express astrocyte markers such as GFAP and S100 and are typically thought of as astrocytes of the gut. The role of enteric glia in gut function is not completely understood, but they are involved in regulating motility via interactions with enteric neurons (McClain et al., 2014). Furthermore, they also play a role in the maintenance of gut epithelial integrity and loss of enteric glia has been shown to lead to intestinal inflammation (Aube et al., 2006). It has also been suggested that enteric glia may influence barrier integrity through interaction with immune cells (Ibiza et al., 2016). The ability of enteric glia to mediate immune responses could be a possible underlying mechanism for Crohn’s disease (Pochard et al., 2018) and they are known to contribute to the inflammation that occurs with conditions of the gut including irritable bowel disease, enterocolitis, and gut infections (von Boyen et al., 2004; Linan-Rico et al., 2016; Lilli et al., 2018). An age-related decrease in the number of enteric glia may play a role in chronic, low-grade inflammation that is associated with age-related gut motility disorders (Franceschi et al., 2007). Treatments for Crohn’s disease are limited and can have serious side-effects (Seyedian et al., 2019). Therefore, gene therapy targeting of enteric glia with neuroprotective strategies in the early stage of such diseases, may be a useful approach to help treat these disorders.

Several studies have looked at the delivery of AAV to the gastrointestinal tract. Oral or enema delivery of AAV serotypes 1–10 have shown transduction of GI tissue including the lamina propria and endothelial cells, but transduction of the ENS was not determined (Shao et al., 2006). When AAV vectors were injected directly into the descending colon, neuronal and enteric glia transduction was observed in the myenteric and submucosal plexuses (Benskey et al., 2015). Of the serotypes tested, AAV serotypes 1, 5, 6, 8, and AAV8-double Y-F + T-V showed both neuronal and glial transduction. However, the authors note that glial transduction was rare for most serotypes except for AAV6, where the level of transduction was roughly equal between neurons and glia. Systemic delivery of AAV serotypes has demonstrated tropism for enteric glia, although few studies have attempted to target glia directly. Both intravenous and intrathecal delivery of AAV9 to juvenile mice leads to transduction of enteric ganglia, but no glial transduction was reported (Schuster et al., 2014). Gombash et al. (2014) showed that intravenous injection of scAAV8 and scAAV9 (which have known tropism for astrocytes) containing a GFP reporter into neonatal and juvenile mice led to GFP expression that was found exclusively in myenteric neurons. GFP expression was occasionally detected in S100 positive glia in neonatal animals for scAAV8. To determine if tropism could be directed toward glia, the authors engineered an AAV9 vector with GFP expression under the control of the *GFAP* promoter, as GFAP expression is present in enteric glia, similar to astrocytes in the CNS. Following intravenous injection of an AAV9-GFAP-GFP vector into neonates, GFP expression was found principally in enteric glia of the myenteric ganglia. However, it should be noted that the number of glial cells transduced was less than 5%. This study also trialed AAV 6, which has been shown to cross the BBB (Zhang et al., 2011) but no transgene expression was detected. This is likely due to differences in vasculature between the ENS and CNS that impact on the virus’s ability to cross the endothelial barrier. In another study (Buckinx et al., 2016) using AAV8 and AAV9 to transduce myenteric and submucosal neurons, about 25–30% of neurons were found to be expressing eGFP. All subtypes of neurons expressed GFP, but no expression was seen in glia (assessed using S100 and GFAP). While it appears that AAV-mediated transgene expression can be somewhat tailored toward enteric glia, further work is required to make this a viable approach to target these cells. One issue is the use of a GFAP promoter and systemic delivery of AAV9, as this would make specific targeting of enteric glia, without potential off-target effects on other GFAP-expressing cells, impossible. Currently, an enteric-glia-specific promoter has not been identified. However, transcriptional profiling of enteric glia suggests that they are developmentally closer to oligodendrocytes and Schwann cells than astrocytes and can adopt some Schwann cell markers (Rao and Gershon, 2018). However, the gut microenvironment shifts these cells toward an astrocyte-like phenotype as shown by expression of GFAP, which is not seen in Schwann cells (Gulbransen and Christofi, 2018). Therefore, enteric glia are considered a novel type of glia, and that an enteric glia-specific promoter may need to be developed. This different phenotype may also explain the low transduction efficiency of enteric glia compared with other glial types. Further testing of novel AAVs would likely be needed to make this a viable approach to therapy.

## Conclusion

Through the use of specific serotypes and cell-specific promoters, glial cell targeting is possible and has shown some promise in experimental models, particularly in the retina. However, there are still several hurdles that need to be overcome for truly glial-specific AAV tropism to be achieved for use in human gene therapy. While several AAV serotypes have increased tropism toward glia, they are not glia-specific, and so any gene therapy transgene will be present in other cell types. Furthermore, the percentage of glial cells that are transduced is also often low, raising the question of whether transgene expression levels would be enough to have a therapeutic effect. The development of promoters that are highly specific for a particular glial cell type would be beneficial to avoid the possibility of off-target effects which might be likely with systemic delivery of an AAV vector expressing transgenes under the control of the GFAP promoter for example, due to it being expressed in a number of glial cell types throughout the body. An issue with the use of cell-specific promoters is that they are often large, which can preclude their use in an AAV vector context. However, a number of studies have demonstrated the use of shortened promoter sequences for driving glial cell-specific transgene expression. The development of new methods for synthetic promoter design holds real promise (Juttner et al., 2019). This study used a number of approaches to design synthetic promoters based on conserved upstream sequences of highly cell-specific genes and cis-regulatory regions active in cell types of interest. This approach led to the discovery of promoters that provide 100% specific gene expression in different retinal cell types, including the 500 bp ProB2 promoter, which shows 100% specificity for Müller cells. Applying this approach to other cell types could lead to the discovery of highly cell-type-specific promoters of appropriate size for use in AAV vectors.

The AAV literature also reports differences in transduction results depending on the model used and the method of delivery. Observations from *in vitro* studies often do not translate to the *in vivo* situation, and differences can be seen depending on the animal species or strain used or the method of delivery. For example, the ability to systemically deliver an AAV vector with a particular cellular tropism or cellular promoter would be beneficial. The variants AAV9-PHP.B and PHP.eB have been reported to allow for significant transduction of the CNS following intravenous infusion but depending on the animal model or method of delivery used, different results are seen (Hordeaux et al., 2018; Simpson et al., 2019). While enhanced CNS tropism has been shown in a number of mouse strains including C57BL/6J mouse where it was first demonstrated, no such increase in transduction efficiency is seen in the BALB/cJ mouse strain (Hordeaux et al., 2018; Matsuzaki et al., 2019). These results are explained by lower levels of the receptor for AAV9-PHP.B, LY6A, in BALB/cJ mice (Huang et al., 2019; Batista et al., 2020). When this virus was delivered to cats, sheep and non-human primates the efficiency of transduction of the CNS was low (Hordeaux et al., 2018; Matsuzaki et al., 2018; Liguore et al., 2019; Batista et al., 2020; Liu et al., 2020) and these species, as well as humans, have no known ortholog for *Ly6a* (Batista et al., 2020). This demonstrates that the use of non-human species to select novel AAV capsid variants could inadvertently limit the usefulness of candidate capsids that are isolated. These differences can also be more subtle. Using the cell-type-specific markers GFAP and Olig-2, He et al. (2019) assessed the cell-specific tropism of AAV serotypes 2, 5, 7, 8, and 9 in C57 and FVB mice. In the case of oligodendrocyte transduction, AAV8 resulted in 23% of oligodendrocytes being EGFP positive in C57 mice, significantly more than was seen measured in FVB mice at only 4.4%. In FVB mice, AAV7 vectors transduced a significantly larger number of oligodendrocytes than AAV8 vectors. Therefore, the choice of model can have a significant impact on the results that are obtained, and it will be important to understand the species and cell-type specific localization of AAV receptors to ensure accurate targeting of AAVs.

In a review by Maes et al. (2019) on the use of gene therapy to target microglia, the authors proposed guidelines for the reporting of viral transduction of this cell type. A similar set of guidelines for design and reporting of glial-targeting gene therapy studies could be of benefit for moving these approaches to clinically useful interventions. In order to confirm the results observed in animals, experiments using human model systems are crucial. While a number of non-human primate studies of AAV tropism have been carried out, these are not practical or financially viable for many research groups to undertake. Studies could be carried out in primary cells derived from the human brain; however, these may not express the same AAV receptors and lack the differentiation of the *in vivo* environment. In order to better understand the tropism and glial cell-specific transduction of AAV vectors, studies using primary human cells as close to their *in vivo* context are needed (Lisowski et al., 2015). One example that has been proposed is the use of humanized mouse models containing chimeric tissues, such as the FRG mouse model that allows animals to be generated with chimeric mouse-human livers (Azuma et al., 2007). While such an approach does not apply to the nervous system, the use of induced pluripotent stem cells (iPSCs) and human brain organoids have potential to understand tropism and expression of transgenes in human cells. Recent studies have used this approach to study AAV transduction and expression in the retina (Mookherjee et al., 2018; Quinn et al., 2019; Tornabene et al., 2019; Garita-Hernandez et al., 2020; Lane et al., 2020). The study by Quinn et al. (2019) specifically looked at the expression in Müller cells within the organoids. Using AAV and ShH10Y445F vectors with either the CMV or RLBP1 promoter showed good Müller cell transduction, with the authors reporting a ShH10Y445F-RLBP1-GFP vector being specific for transduction in Müller cells. A similar tropism and expression potency was seen in cultured adult human retinal explants. This is an important observation, as it demonstrates findings seen in organoids may recapitulate what would be seen in adult human tissue. Studies have also used human iPSC-derived cerebral organoids to study AAV transduction (Latour et al., 2019; Depla et al., 2020). However, these studies only looked at neuronal transgene expression achieved from the AAV vectors used. While organoids are likely useful for testing AAV for treating developmental disorders, using optimal differentiation conditions enables the creation of cerebral organoids containing mature neurons and astrocytes (Yakoub, 2019) that could be used to study AAV glial tropism. Models of human intestinal organoids that contain an ENS (including glia) could potentially be used for this purpose (Schlieve et al., 2017).

## Author Contributions

SO’C, WC, and DY wrote the manuscript. All authors contributed to the article and approved the submitted version.

## Conflict of Interest

The authors declare that the research was conducted in the absence of any commercial or financial relationships that could be construed as a potential conflict of interest.
